# A psychometric evaluation of the Musculoskeletal Health Questionnaire (MSK-HQ): validation and measurement invariance in inflammatory arthritis

**DOI:** 10.1093/rap/rkaf041

**Published:** 2025-04-11

**Authors:** Nikita Arumalla, James B Galloway, Joanna Ledingham, Toby Garrood, Sam Norton

**Affiliations:** Faculty of Life Sciences and Medicine, Academic Rheumatology, King’s College London, London, UK; Faculty of Life Sciences and Medicine, Academic Rheumatology, King’s College London, London, UK; Rheumatology, Queen Alexandra Hospital, Portsmouth, UK; Rheumatology, Guy’s and St Thomas’ NHS Trust, London, UK; Faculty of Life Sciences and Medicine, Academic Rheumatology, King’s College London, London, UK

**Keywords:** inflammatory arthritis, patient-reported outcome measures, musculoskeletal health, quality of life

## Abstract

**Objectives:**

The Musculoskeletal Health Questionnaire (MSK-HQ) is a patient reported outcome measure (PROM) co-produced for use across musculoskeletal diseases. This study analyses the validity, reliability, sensitivity to change and measurement invariance of the MSK-HQ in inflammatory arthritis (IA).

**Methods:**

A total of 5106 patients recruited to the National Early Inflammatory Arthritis Audit (NEIAA) between May 2018 and March 2020 with a diagnosis of IA were included. Patients completed PROMs at baseline and 3 and 12 months alongside clinic visits. Convergent validity was assessed in relation to the HAQ-II, Patient Health Questionnaire 4 (PHQ-4) and 28-item DAS (DAS28). Construct validity was assessed using confirmatory factor analysis (CFA). Hierarchical tests for configural, metric and scalar invariance determined measurement invariance in item CFAs.

**Results:**

The MSK-HQ total score correlated well with the HAQ-II (*r* = −0.79) and PHQ-4 (*r* = −0.66) and moderately with the DAS28 (*r* = −0.42). A unidimensional structure for the MSK-HQ was confirmed only when two items relating to illness perception were excluded. The MSK-HQ total score demonstrated good sensitivity to change. Reliability was high (α = 0.93). The minimum clinically important difference was 4 points across the IA subtypes. Significance was noted in tests of DIF for a few MSK-HQ items, but the level of bias was small.

**Conclusion:**

This study provides evidence for the validity and sensitivity to change of the MSK-HQ in patients with IA, with a change of >4 points likely to be clinically meaningful. The MSK-HQ has high convergent and construct validity and is sensitive to change, providing a valuable tool for clinical care and research studies.

Key messages

- The MSK-HQ is valid and sensitive to change when assessing musculoskeletal health in inflammatory arthritis.
- The MSK-HQ can be used across the inflammatory arthritis subtypes.
- A change of >4 points in the MSK-HQ score is likely to be clinically meaningful in patients with inflammatory arthritis.

## Introduction

Patient-reported outcome measures (PROMs), such as the Health Assessment Questionnaire (HAQ) [1] and the EuroQol 5-Dimensions (EQ-5D) [2], are widely used in healthcare settings to capture aspects of health-related quality of life. These tools are particularly useful in research using a nomothetic approach, particularly where outcomes need to be considered across different conditions (e.g. in clinical trials and health economic modelling of treatment effects). However, they are sometimes criticised in terms of their relevance to the patient and their utility for decision-making for specific individuals in the clinic [3].

The Musculoskeletal Health-related Quality of Life (MSK-HQ) PROM was developed using a co-production approach to be applicable across a range of musculoskeletal diseases with maximal content validity [4]. The tool incorporates assessment of somatic symptoms such as pain, stiffness and fatigue, as well as disease impact on daily activities, psychological well-being, understanding of disease and self-management ability. The MSK-HQ has been validated in musculoskeletal conditions across various patient cohorts, including orthopaedics clinics, community physiotherapy and primary care [5, 6]. In an inflammatory arthritis (IA) cohort, its psychometric properties compared well with the HAQ, EQ-5D and Rheumatoid Arthritis Impact of Disease (RAID) in patients with RA and the Psoriatic Arthritis Impact of Disease (PsAID) in patients with PsA [6, 7]. However, detailed information on sensitivity to change has not previously been studied in IA and a minimum clinically important difference (MCID) has not been defined in this population. Furthermore, research has not confirmed whether the MSK-HQ is valid in terms of measurement equivalence across IA subtypes.

A key assumption for any PROM is that of measurement equivalence [8]; that is, the measurement properties (e.g. discrimination and difficulty parameters) are equivalent across groups and thus scores on the PROM are comparable across these groups. The change in clinical and patient-reported measures over time is frequently used to assess treatment effectiveness in patients with IA, based on an assumption that genuine changes in patient symptoms are reflected by changes in raw scores. With the MSK-HQ PROM that was developed to be pertinent across a range of musculoskeletal diseases, we need to be able to meaningfully interpret and compare scores across the various IA subtypes. If the score change does not accurately reflect true clinical change or is not comparable between types of IA, then differences in the MSK-HQ item score between these diseases may not accurately reflect actual symptom differences and could simply be due to measurement error. It is important to determine whether the scale remains functionally unchanged across all types of IA to allow valid comparisons across these disease groups. Measurement equivalence testing can evaluate if any differences observed are true or impacted by item bias where individual questions in the PROM can have different meanings or relevance for patients with different types of IA, or response bias where patients in these groups have varying styles of response to the same PROM items [9].

In 2018, the Health Quality Improvement Partnership (HQIP) commissioned a national audit of the care of IA, which incorporated routine collection of the MSK-HQ [10]. Content validity of the MSK-HQ has been reported to be high [4]; we now wish to assess construct validity of the MSK-HQ and concurrent validity with the HAQ-II [11], which is an established measure of function in patients with musculoskeletal diseases. Previous validation studies have been based on relatively small samples that have not been sufficiently powered to detect measurement invariance. Furthermore, the National Early Inflammatory Arthritis Audit (NEIAA), with a baseline assessment early in the disease course prior to treatment initiation, allows for a robust estimate of the MCID to be generated for early IA (EIA) populations.

Our aim was to use the NEIAA dataset to further describe the psychometric properties of the MSK-HQ, including its validity, measurement invariance across disease subtypes and sensitivity to change and to define an MCID in RA.

## Methods

### Study sample

The NEIAA captures data on patients referred to rheumatology services in England and Wales with suspected EIA. Its primary goal is to assess care quality across healthcare providers. It launched on 8 May 2018 and collected information on patients ≥16 years of age referred with suspected IA [including RA, PsA and peripheral axial SpA (axSpA)]. For this study we included patients enrolled between 8 May 2018 and 1 March 2020 receiving a diagnosis of RA, PsA, undifferentiated IA (UIA) or axSpA by their treating rheumatologist. Upon patient enrolment, eligible patients who had received an IA diagnosis were sent an e-mail link to complete a series of PROMs. Patients also had the option of completing paper forms for their clinical team to upload. If no form was returned, a single electronic reminder was sent 2 weeks later. Clinical information was collected at baseline, 3 months after diagnosis and again at 12 months after diagnosis, with PROMs requested contemporaneously to these clinical visits.

The present analysis included any patient receiving a diagnosis of RA, PsA, UIA or axSpA and returning a baseline PROM and having responded to at least half of the MSK-HQ items. Overall, 13 129 people between 8 May 2018 and 1 March 2020 received a diagnosis of IA. Of which 5440 (41%) returned a baseline MSK-HQ. A further 87 were excluded due to completing less than half of the MSK-HQ items and 247 for having a diagnosis of an IA subtype not considered in this analysis.

### Variables

Sociodemographic characteristics captured at baseline included age, gender, ethnicity (White, Black British: African or Caribbean, Asian or Asian British or Other). The patient-reported questionnaires included the MSK-HQ, HAQ-II, Patient Health Questionnaire 2 (PHQ-2) and Generalised Anxiety Disorder 2 (GAD-2) and with disease activity assessed using the 28-item DAS (DAS28), which includes counts of swelling and tenderness of 28 joints, CRP and a patient global assessment of disease activity on a 0–100 scale.

The MSK-HQ contains 15 items. The first 14 items relate to facets of musculoskeletal health including pain and stiffness, physical function, impact on work and social activities, sleep and fatigue, emotional impact and understanding of disease and self-management ability. The 15th item captures physical activity levels in the past week and is not part of the total scoring; thus it was not included in our analysis. The first 14 items are scored on a 0–4 scale, with the total MSK-HQ derived from summation of the 14 items; scores range between 0 and 56, with higher scores indicating better outcomes [4].

The HAQ-II is a measure of functional ability in musculoskeletal disease, assessing the level of difficulty in completing specific activities of daily living. This is a revised 10-item version of the HAQ, with 9 items assessing functional limitations and 1 item assessing the ability to do outside work, as a measure of disability. Scores range between 0 and 3, with lower scores indicating better outcomes [11].

Mental health was assessed in the national audit dataset using two questionnaires, the PHQ-2 and GAD-2, validated in the assessment of depression and anxiety, respectively. Each measure contains two items, with the score of each measure ranging from 0 to 6; lower scores indicate lower levels of psychological distress [12, 13]. Given the high degree of overlap between symptoms in people with anxiety and depression, the PHQ-2 and GAD-2 can be combined to generate a score with a range of 0–12 indicating general psychological distress, which acts as a screen for both depression and anxiety, referred to as the PHQ-4 [14].

### Ethical approval

Approval to conduct this study was obtained from the HQIP. Informed consent was not required, as the Secretary of State for Health has granted permission to the NEIAA to collect data for the purpose of national audits. Secondary uses of this anonymous data for the use of research have been separately approved (Clinical Advisory Group: 19/CAG/0059, Research Ethics Committee reference: 19/EE/0082, 6 December 2019). Data access requests can be made through the HQIP and are subject to a data sharing agreement.

### Statistical analysis

To assess dimensionality, the number of latent variables underlying the interitem correlation matrix (i.e. the number of underlying factors that explain relationships between items), we used principal components analysis (PCA) combined with parallel analysis (PA). Given the ordinal response format, polychoric correlations were estimated. Construct validity was then assessed using confirmatory factor analysis (CFA), which is used to test whether the MSK-HQ items align with the expected factor structure. Specifically, item CFA models were estimated via full information maximum likelihood with an ordered logistic approach. Factor structure refers to how the different MSK-HQ items group together to measure underlying concepts that are not directly observable (i.e. pain, fatigue). It is useful to note that this model is equivalent to the item response theory (IRT) graded response model [15]. To aid interpretation, discrimination parameters (i.e. factor loadings) were transformed to a correlation metric, where the loadings indicate the standardized association between the underlying latent variable and the item (i.e. higher values indicate stronger associations between the item and the underlying construct). Difficulty parameters (i.e. thresholds) were retained on the original metric and indicate the expected level of the underlying latent trait where 50% of people would endorse the respective response level or higher.

Determining measurement equivalence for item CFAs involves a series of hierarchical tests: configural invariance, metric invariance and scalar invariance. Respectively, this tests that the scale assesses the same underlying latent construct consistently across different subgroups. Configural invariance was determined via PCA and PA in each group. Metric and scalar invariance were tested using likelihood ratio tests for multiple group CFAs where loadings (metric) and loadings and thresholds (scalar) were constrained to be the same across groups, against a non-invariant model where these parameters were freely estimated across groups. Given the large sample size and number of tests involved, the critical α level for concluding significant invariance was set at *P* < 0.001. Furthermore, model comparisons were considered using the Bayesian information criterion (BIC), where lower values indicate better model fit, balancing model complexity and goodness of fit.

Multiple approaches to calculating MCIDs exist and there is no clear consensus on the optimal approach. Given the lack of information to use an anchor-based approach, we estimated MCIDs using two distributional approaches: the s.e. of the measurement and one-third of a s.d. [16]. In addition to estimates of the MCID, the standardized response mean between 0 and 12 months was calculated by dividing the mean change in 0–12 months by the s.d. of the change.

All analyses were undertaken in Stata 17.0 (StataCorp, College Station, TX, USA). A glossary of statistical terms is available in Supplementary Fig. S1, available at *Rheumatology Advances in Practice* online.

## Results

### Sample characteristics

A total of 5106 people met the inclusion criteria. Of these, 73% were considered to have RA, 13% PsA, 2% axSpA and 12% UIA. Demographic and clinical characteristics at baseline are presented in Table 1. The mean age was 58 years, 62% were female and 91% were White British, European or other ethnicity.

**Table 1.** Baseline descriptive characteristics

Characteristics	Total (*N* = 5106)	RA (*n* = 3745)	PsA (*n* = 648)	axSpA (*n* = 117)	UA (*n* = 596)
Age, years, mean (s.d.)	57.6 (15.8)	59.9 (14.9)	49.6 (14.8)	36.9 (11.6)	56.0 (16.8)
Female, *n* (%)					
Yes	3167 (62.0)	2378 (63.5)	368 (56.8)	48 (41.0)	373 (62.6)
No	1939 (38.0)	1367 (36.5)	280 (43.2)	69 (59.0)	223 (37.4)
Ethnicity, *n* (%)					
White	4669 (91.4)	3412 (91.1)	602 (92.9)	108 (92.3)	547 (91.8)
Black	72 (1.4)	62 (1.7)	<11	<11	<11
Asian	238 (4.7)	169 (4.5)	32 (4.9)	<11	–^a^
Mixed	18 (0.4)	14 (0.4)	<11	<11	<11
Other	81 (1.6)	66 (1.8)	<11	<11	<11
Not known	28 (0.5)	22 (0.6)	<11	<11	<11
Baseline DAS28, mean (s.d.)	4.7 (1.5)	4.8 (1.4)	4.2 (1.4)	2.8 (1.3)	4.2 (1.4)
HAQ-II disability (0–3), mean (s.d.)	1.1 (0.7)	1.2 (0.7)	1.0 (0.7)	1.0 (0.6)	1.0 (0.7)
PHQ-4ADS distress (0–12), mean (s.d.)	4.6 (3.9)	4.7 (3.9)	4.4 (3.9)	4.7 (3.6)	4.5 (3.9)
MSK-HQ total score (0–56), mean (s.d.)	25.6 (11.4)	25.4 (11.4)	26.8 (11.1)	25.6 (11.4)	26.0 (11.3)

a Redacted due to low number suppression.

### Item and total score characteristics

In the total sample, all items demonstrated responses across the full range of possible response options with no major indication of floor or ceiling effects at the item level (Supplementary Fig. S2, available at *Rheumatology Advances in Practice* online). Similarly, the MSK-HQ total score demonstrated a wide range of scores that were approximately normally distributed and with no indication of a problem with floor or ceiling effects (Supplementary Fig. S3, available at *Rheumatology Advances in Practice* online).

**Figure 1.**
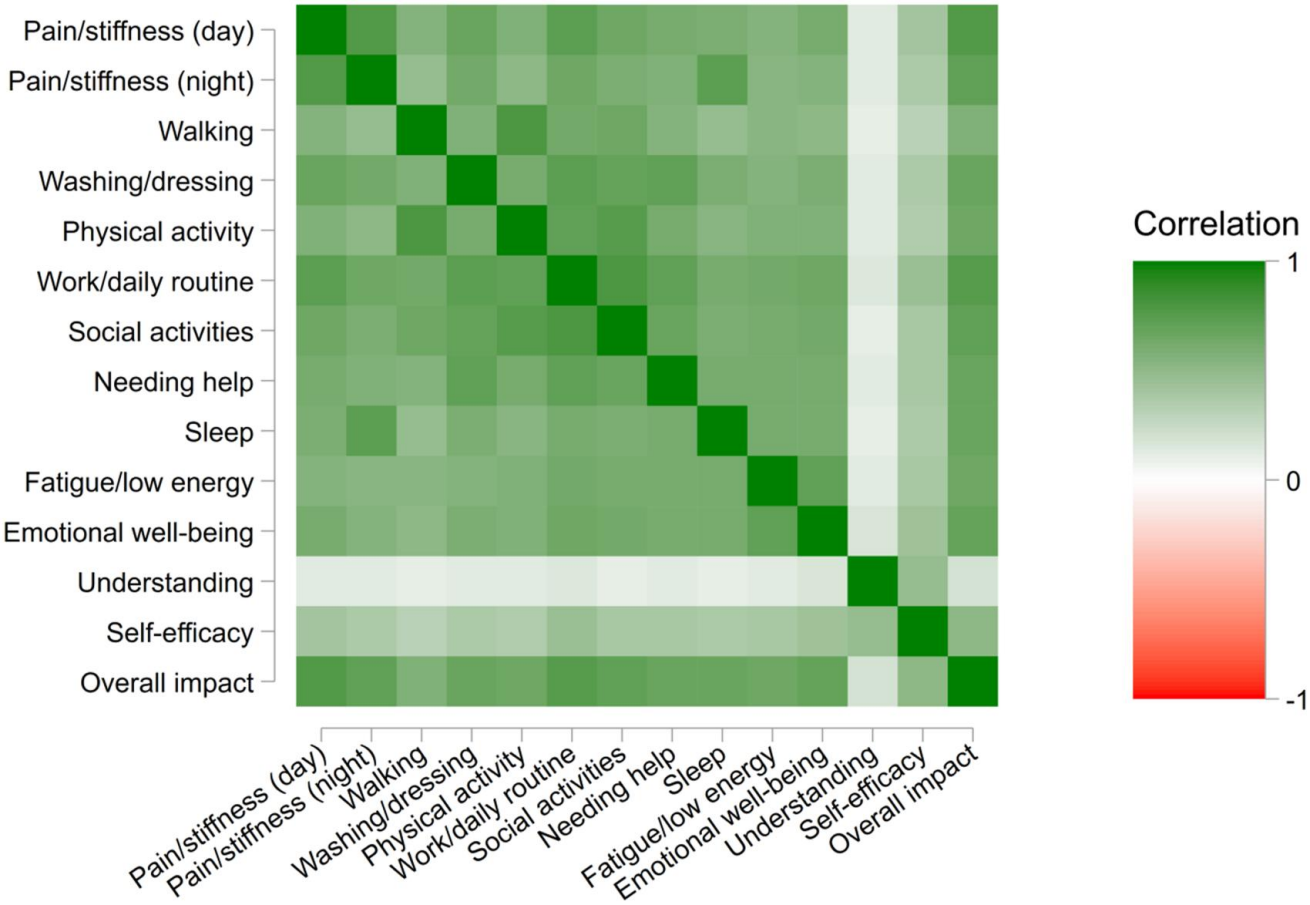
Item polychoric correlation heat map. This figure shows the strength of relationships between each pair of MSK-HQ items, where darker colours indicate stronger correlations

Interitem correlations for the 14 MSK-HQ items (Fig. 1, Supplementary Fig. S4, available at *Rheumatology Advances in Practice* online) were all positive and generally considered to be strong, except for the understanding and self-efficacy items, which while being positive, were of lower magnitude.

**Figure 2.**
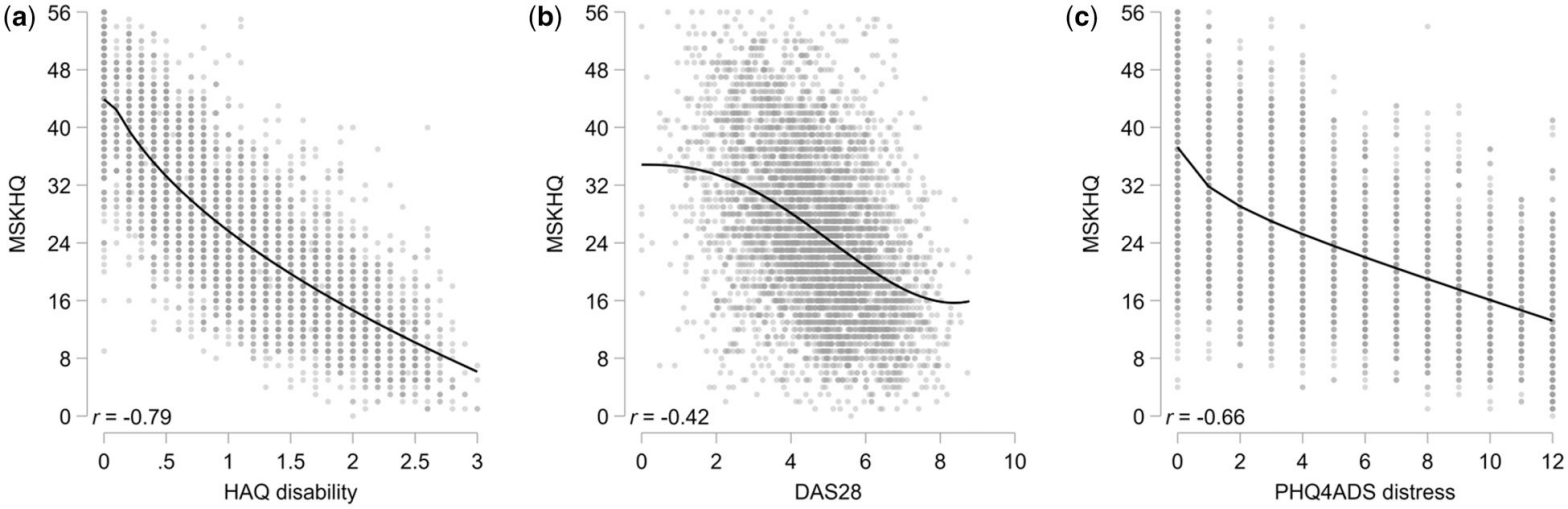
Convergent validity of the MSK-HQ against **(a)** disability, **(b)** disease activity and **(c)** psychological distress. Overlaid lines are fractional polynomial smoothers indicating linearity of association

Convergent validity of the total score against measures of disability, disease activity and psychological distress was good (Fig. 2, Supplementary Fig. S5, available at *Rheumatology Advances in Practice* online). For each of the variables there was an indication of non-linearity of the association. For disability and psychological distress, this was due to floor effects for the HAQ-II and PHQ-4 Anxiety-Depression Scale (PHQ-4ADS), indicating that the MSK-HQ was better at capturing differences between those scoring at the lowest levels of those variables. For the DAS28, however, the indication was that the MSK-HQ was less able to discriminate between very low (<2) or high (>7) levels of disease activity.

### Construct validity and reliability

**Figure 3.**
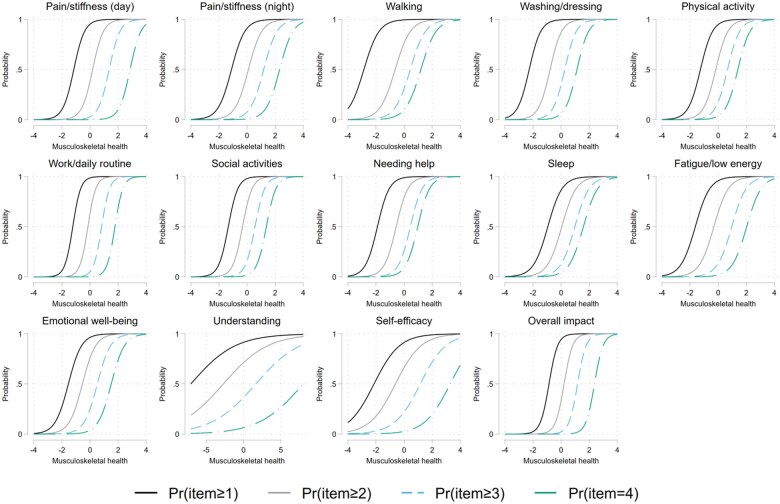
Item characteristics curves. This figure shows how the probability of selecting each response for MSK-HQ items changes across levels of the underlying trait (i.e. pain, function). Each curve represents the likelihood of endorsing higher response categories, helping us understand how well each item can distinguish between different levels of the trait

Parallel analysis indicated a two-factor structure with one dominant first factor and a small secondary factor. Estimation of a unidimensional model indicated that item 12 regarding understanding and item 13 regarding self-efficacy as having considerably lower loadings, correlated at 0.18 and 0.52 with the underlying construct compared with loadings >0.7 for all other items (Supplementary Fig. S6, available at *Rheumatology Advances in Practice* online). This can also be seen in the item characteristics curves (Fig. 3), where the slopes of the curves are much flatter, indicting poorer discrimination. Fitting a two-factor model with items 12 and 13 loading onto a second factor indicated better fit to the data than a unidimensional model (BIC 169 157 *vs* 169 545, respectively), although the factor loadings for items 12 and 13 needed to be constrained to be equal for identification. Further analysis using a bifactor model with a general factor and two subgroup factors further supported a two-factor structure, as the loadings of items 12 and 13 on the general factor were low. Taken together, there is strong support for a unidimensional structure only when items 12 and 13 are excluded.

A test characteristics curve measuring the relationship between the observed MSK-HQ total score and the latent variable demonstrated some evidence of non-linearity in the extremes of scoring, suggesting the total score is less reliable in those with extremely good or extremely poor musculoskeletal health (Supplementary Fig. S7, available at *Rheumatology Advances in Practice* online). This was confirmed by the test information function (Supplementary Fig. S8, available at *Rheumatology Advances in Practice* online). In IRT, the concept of information is closely related to the s.e. of the measurement, and thus to reliability, and allows evaluation of scale precision across the range of the latent variable. Test information demonstrated high reliability (>0.8) between ≈3 s.d.s either side of the mean of the latent measure of musculoskeletal health. This was corroborated by a Cronbach’s α value of 0.93 (Table 2).

**Table 2.** Means, s.d.s, reliability and MCID estimates for full MSK-HQ based on 12-month visit (full scale, range 0–56)

Working diagnosis	*n*	Mean	s.d.	α	MCID_S.E.M._	MCID_⅓S.D._
RA	1283	37.5	12.3	0.93	3.9	4.1
PsA	188	35.3	13.3	0.93	4.2	4.4
axSpA	17	35.2	12.4	0.93	3.9	4.1
UIA	175	37.3	12.4	0.92	3.9	4.1
Total	1663	37.2	12.4	0.93	3.9	4.1

### Measurement invariance

Measurement invariance was considered by disease subtype. Tests for configural invariance indicated that a general factor explained the majority of the variance between items, with some indication of a weak second factor capturing items 12 and 13 in all the IA subtypes.

**Figure 4.**
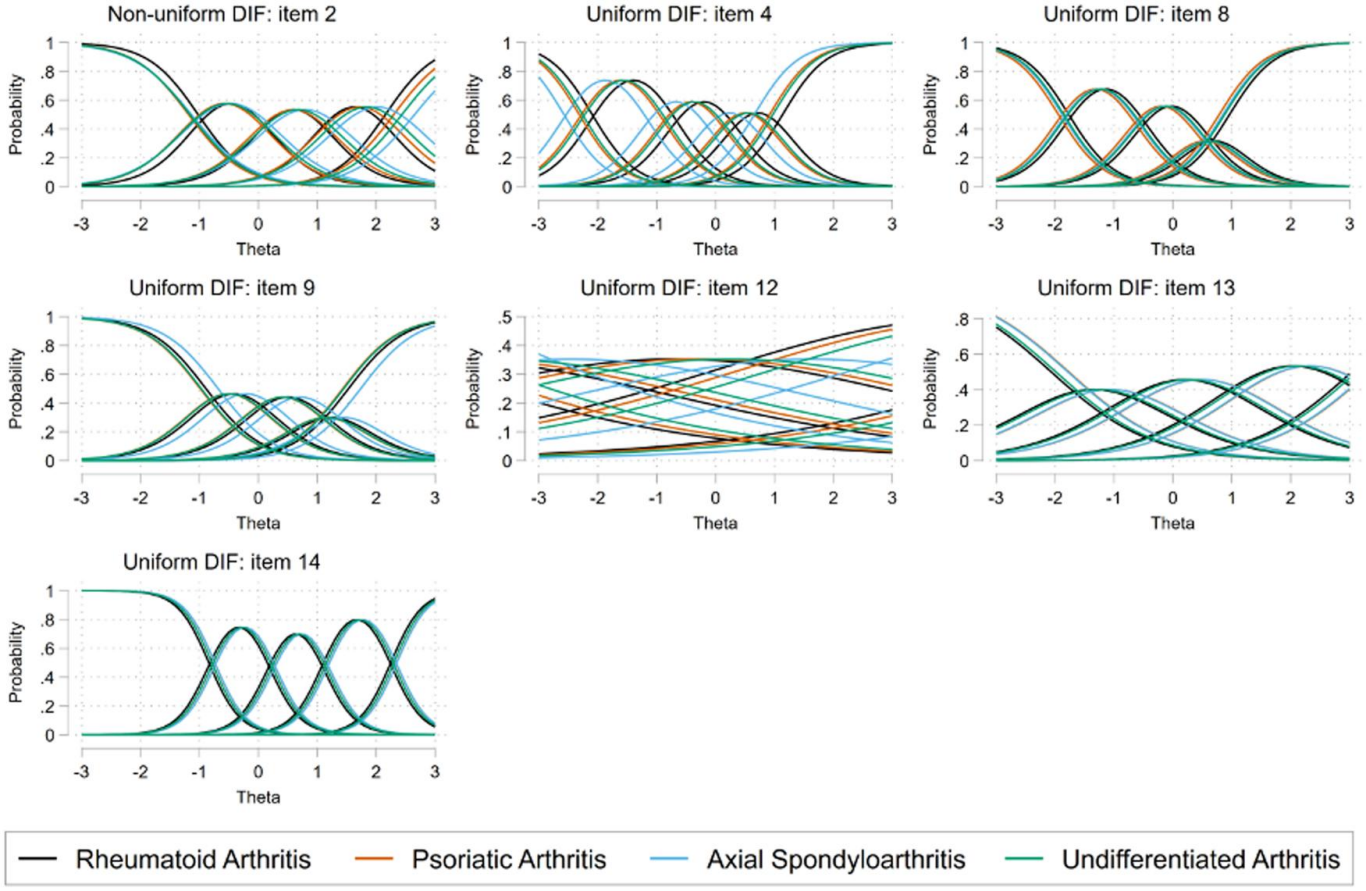
DIF analysis of MSK-HQ items across the IA subtypes, with the *x*-axis representing the level of the musculoskeletal health latent variable (θ) and the *y*-axis representing the probability or likelihood of selecting a specific item response option at specific levels of the musculoskeletal health latent trait. Thus this figure shows how MSK-HQ items perform across different levels of IA, assessing if any items behave differently for each subtype

However, tests indicated issues with assumptions of both metric and scalar invariance: metric χ^2^(42) = 63.2 and *P* = 0.019; scalar χ^2^(209) = 516.2, *P* < 0.001. Further examination of individual items indicated significant non-uniform differential item functioning (DIF) for item 2 (night-time pain) and significant uniform DIF for items 4 (washing/dressing), 8 (needing help), 9 (sleep), 12 (understanding), 13 (self-efficacy) and 14 (overall impact) (Fig. 4). The level of bias introduced appears to be relatively small and unlikely to influence interpretations of the total score (Supplementary Fig. S9, available at *Rheumatology Advances in Practice* online). Significant uniform DIF was still observed for items 4, 8, 9 and 14 in a sensitivity analysis removing items 12 and 13 from the models.

### Responsiveness

Responsiveness was considered for 1663 (33%) people who provided follow-up data at 12 months. The MSK-HQ total score demonstrated good sensitivity to change, with a large effect size for change between baseline and 12 months [standardized mean response 1.01 (95% CI 0.95, 1.07)]. This compares well against the DAS28 [−1.23 (95% CI −1.29, −1.17)] and is stronger than both the HAQ-II [−0.62 (95% CI −0.68, −0.56)] and PHQ-4ADS [−0.57 (95% CI −0.63, −0.51)]. The correlation between the change in MSK-HQ total score was strong with the change in HAQ-II disability (*r* = −0.75) and moderate with the change in DAS28 disease activity and PHQ-4ADS distress (*r* = −0.45 and −0.54, respectively) (Supplementary Fig. S10, available at *Rheumatology Advances in Practice* online).

The MCID calculated based on the s.e.m. for the overall sample was 3.9, where the s.d. is 12.4 based on the 12-month assessment and reliability conservatively assumed to be 0.9. This agrees with a further distributional estimate of MCID of one-third of a s.d. and is consistent across IA subtypes (Table 2). Given that the MSK-HQ scoring does not use fractional numbers, it is sensible to round to the nearest integer, giving an MCID estimate of 4 points across RA, PsA, axSpA and UA subtypes. Repeating the analysis with the reduced 12-item scale gave an identical number when rounded to the nearest integer (Supplementary Fig. S11, available at *Rheumatology Advances in Practice* online).

## Discussion

The MSK-HQ was co-developed by patients and clinicians to measure quality of life related to musculoskeletal health, regardless of the type of musculoskeletal disease or care pathway the patient is on. Our study has reaffirmed the performance of this PROM in assessing this measure in patients with IA and that its psychometric properties are acceptable. There was good correlation between the MSK-HQ and HAQ-II and PHQ-4ADS, indicating the MSK-HQ is well related to other metrics of functional ability and psychological distress, respectively. The MSK-HQ was superior in assessing differences in health in patients with mild disease, as there was no floor effect apparent here, unlike the HAQ-II and PHQ-4ADS. However, relative to the clinician assessed DAS28, the MSK-HQ seems less able to differentiate between those with very low or very highly active disease (DAS28 <2 or >7). We recognize that the DAS28 is primarily validated in RA but was also the disease activity measure captured in all patients in the NEIAA. The DAS28 may not fully capture disease activity in axSpA, which likely explains the lower mean scores observed in this subgroup. However, given that axSpA represented a very small proportion of our total cohort (2.3%), any potential bias introduced is unlikely to have had any meaningful impact on the overall findings.

While the MSK-HQ is a multidimensional measure using a formative measurement model, practical scoring using a unidimensionality approach has been suggested due to high internal consistency across the scale items in the initial cohort, with a Cronbach’s α of 0.88 [4]. However, further validation in an IA cohort of 287 patients showed weaker loadings for items 12 and 13 assessing understanding (0.19) and self-efficacy (0.52) [6]. The IRT graded response model in our study has confirmed this, with items 12 and 13 loading 0.18 and 0.52, respectively, *vs* >0.7 for all other items.

Additional analyses confirmed that these two items pertaining to illness perceptions behave differently than the other items and likely assess a different latent construct. Illness perception in IA is associated with pain, function, psychological health and medication adherence [17–20]. In the development of the MSK-HQ PROM, patients indicated they found all questions highly relevant to their lives and decided against a weighted approach in scoring this tool. Overall, the understanding of disease and confidence in self-managing that disease is of clinical benefit, but these measures are not necessarily related to disease severity. Using the full 14-item scale might not be sufficiently reliable for use in clinical research or trials, with the 12-item scale more judicious to use. However, in routine clinical use, items 12 and 13 are likely to support health understanding and behaviour, positively influencing health outcomes, although full consideration of illness perceptions could be assessed using scales such as the Brief Illness Perceptions Questionnaire [21].

In the analysis of measurement invariance of the MSK-HQ, configural invariance was established; a two-factor latent structure with a dominant primary factor and weaker secondary factor was consistent across the IA subtypes. However, there was a lack of metric and scalar invariance between the groups and thus the MSK-HQ is not measurement invariant by IA subtype. In other words, some of the MSK-HQ items did not measure the latent trait of musculoskeletal health in the same way in each disease type. AxSpA seemed most different from the other IA subtypes. This may be reflective of the disease phenotype, where patients with axSpA have mainly spinal disease, as opposed to peripheral disease with the other types of IA; for instance, items 4 and 8 (relating to washing and dressing and needing help, respectively) may perform differently in those with pain and stiffness symptoms in the back *vs* the limbs. Furthermore, item 2, regarding pain during the night, was less indicative of overall musculoskeletal health. Overall, despite the statistical significance seen with tests of DIF, the level of bias is small and will not impact the interpretations of the total MSK-HQ score.

Minimally important change in the initial validation cohort of patients with musculoskeletal disease from community physiotherapy and orthopaedics was reported to be 5.5 (95% CI 2.7, 8.3), determined by receiver operating characteristics curve analysis [22]. Our study showed that in an IA cohort, a slightly smaller MSK-HQ score change of 4 is a clinically important change, both using the full MSK-HQ total (full scale range 0–56) or the 12-item scale (items 1–11 and 14, full scale range 0–48). The difference between these estimates may be due to several factors, including purely measurement error, but could also include differences in the methods used, plus variation in cohorts and their overall health, length of follow-up and interventions used between instrument testing [23, 24]. Due to the type of conditions seen routinely in an orthopaedics department, it is likely the majority of patients in the initial MSK-HQ validation study had osteoarthritis, a non-inflammatory musculoskeletal disease.

The use of a large national dataset with all patients early in the course of their disease is a strength of our study. Limitations include a sample that was largely patients with RA, and so the generalizability across the other IA types warrants further exploration, especially in axSpA where the cohort represented <5% of the total sample.

The MSK-HQ has now become an established outcome measure for use in clinical practice in rheumatology care. Our study has further substantiated the validity, reliability and sensitivity to change of the MSK-HQ PROM in an IA cohort. For inflammatory rheumatic diseases, a change of >4 points is likely to be clinically meaningful. The MSK-HQ is sensitive to change, and acknowledging the previously described high face validity to patients, it also represents a valuable tool for research studies.

## Supplementary Material

rkaf041_Supplementary_Data

## Data Availability

The data underlying this article were provided by the HQIP by data access request. Any request for these data must be made through the HQIP and are subject to a data sharing agreement.
