# Supplementary material for: A psychometric evaluation of the Musculoskeletal Health Questionnaire (MSK-HQ): validation and measurement invariance in inflammatory arthritis
Source: Rheumatol Adv Pract. 2025 Apr 11;9(2):rkaf041. doi: 10.1093/rap/rkaf041 (PMC12064166; doi:10.1093/rap/rkaf041)
Supplement: rkaf041_Supplementary_Data [file rkaf041_supplementary_data.docx]

**Supplementary data**

| Statistical term | Explanation |
| --- | --- |
| Bayesian information criterion | A statistical measure used to assess the fit of a model whilst taking model complexity into account. A lower value indicated a better balance of model fit and complexity. |
| Ceiling effect | A measurement issue that occurs when too high a percentage of participants score near the maximum possible score. This can make it difficult to detect differences in higher levels of the trait being measured (i.e. pain). |
| Configural invariance | This refers to the concept that a questionnaire should assess the same underlying concept across different groups, without needing all the relationships between items to be the same across groups. i.e. it assesses if the MSKHQ measures musculoskeletal health in a similar way for patients with different types of inflammatory arthritis. |
| Confirmatory factor analysis | A statistical technique that examines the relationship between observed variables (i.e. MSKHQ questions) and their underlying latent constructs (i.e. pain, function). |
| Construct validity | This refers to how well a tool or questionnaire measures the concept it is intended to measure. |
| Convergent validity | This is a measure of how well a tool or measurement correlates to other tools or measurements that assess the same construct. |
| Differential item functioning | This is a statistical analysis that identifies when a test item performs differently for different groups. |
| Dimensionality | This refers to the number of underlying concepts that a set of items on a questionnaire measures; if it measures only one underlying concept (i.e. pain), the questionnaire is unidimensional. |
| Factor loadings | This refers to the strength of the relationship between each item on the questionnaire and the underlying concept it is meant to measure. |
| Factor structure | This refers to how different questionnaire items group together to measure underlying concepts that are not directly observable (i.e. pain, fatigue). |
| Floor effect | A measurement issue that occurs when too high a percentage of participants score near the lowest possible score. This can happen if there is a limited range at the lower end of the scale, and makes it difficult to detect differences in lower levels of the trait being measured (i.e. pain). |
| Inter-item correlation | A measure that assesses how related the scores are between items on a scale. It is a measure of internal consistency of a tool. |
| Measurement invariance | This ensures that a tool or questionnaire measures the same underlying concept consistently across different groups. |
| Metric invariance | This tests if the strength of association between each questionnaire item and the underlying concept are consistent for different groups. |
| Minimum clinically important difference | This relates to the smallest change in a patient’s score on a questionnaire that is perceived as meaningful by the patient or clinician. |
| Parallel analysis | A statistical technique used to determine the number of underlying factors in a set of data, and helps determine the number of components to keep in a principal components analysis. |
| Polychoric correlation | A method used to assess the relationship between two ordinal variables, used when the data is not continuous but still used to represent an underlying continuous trait i.e. pain. |
| Principal components analysis | A dimensionality reducing method used to reduce the complexity of a large data set by identifying the most important underlying patterns. |
| Reliability | Reliability assesses whether a tool is able to produce consistent and reproducible results, and is free from error as much as possible. |
| Responsiveness | Sensitivity to change is the ability of an instrument to measure a change in state, and responsiveness refers to an instrument’s ability to detect clinically meaningful change over time e.g. in response to treatment |
| Scalar invariance | This ensures that the item thresholds at which responses change are the same across groups; i.e. it tests if response interpretations (i.e. mild, moderate and severe) are consistent across groups. |

Supplementary Figure S1: Glossary of statistical terms


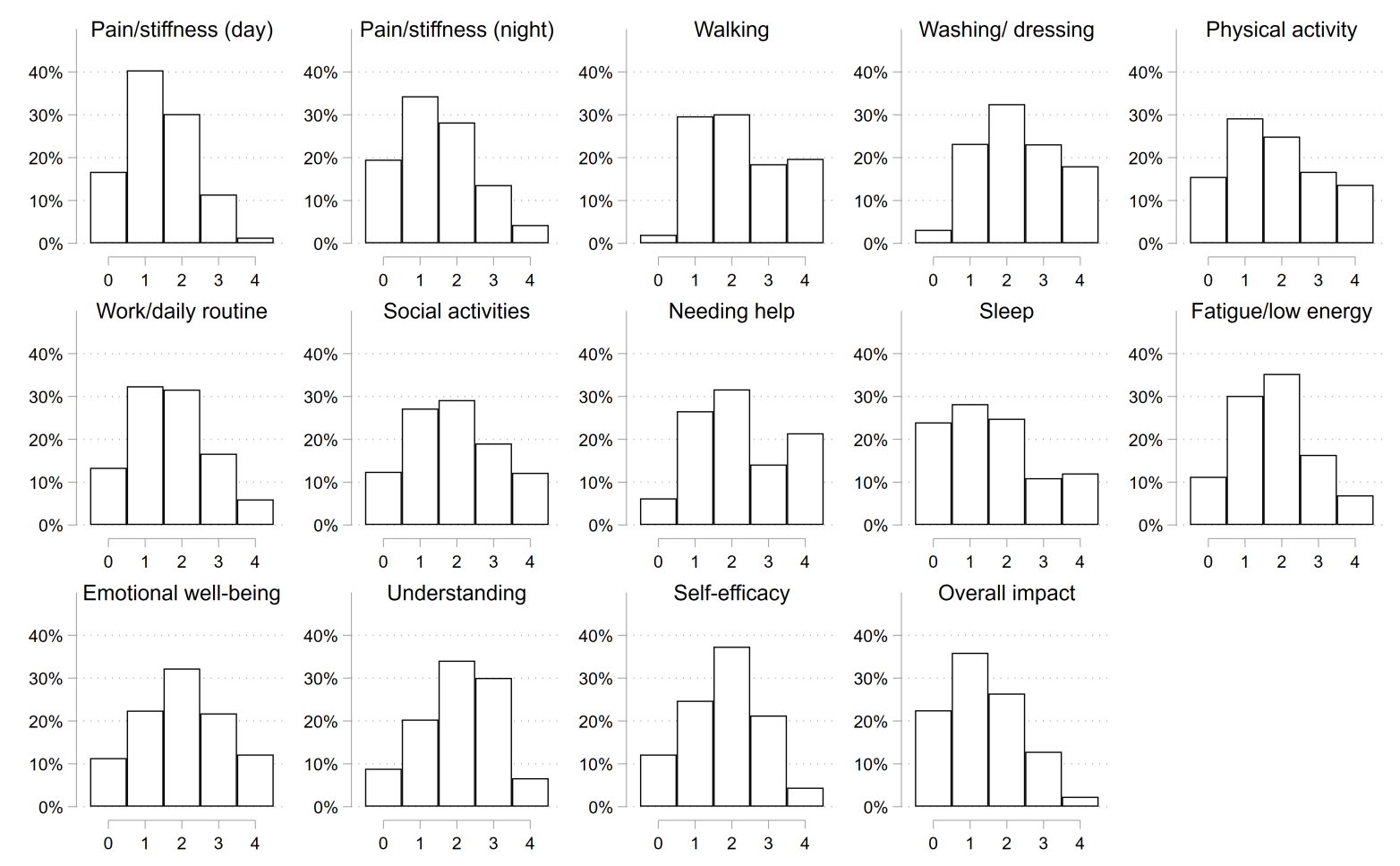


Supplementary Figure S2: Item response distributions of the individual MSKHQ items

Supplementary Figure S3: Distribution of the MSKHQ total score

| Item | Q1 | Q2 | Q3 | Q4 | Q5 | Q6 | Q7 | Q8 | Q9 | Q10 | Q11 | Q12 | Q13 | Q14 |
| --- | --- | --- | --- | --- | --- | --- | --- | --- | --- | --- | --- | --- | --- | --- |
| Q1. Pain/stiffness in the day | 1.00 |  |  |  |  |  |  |  |  |  |  |  |  |  |
| Q2. Pain/stiffness at night | 0.78 | 1.00 |  |  |  |  |  |  |  |  |  |  |  |  |
| Q3. Walking | 0.56 | 0.49 | 1.00 |  |  |  |  |  |  |  |  |  |  |  |
| Q4. Washing/dressing | 0.67 | 0.63 | 0.56 | 1.00 |  |  |  |  |  |  |  |  |  |  |
| Q5. Physical activity | 0.58 | 0.53 | 0.79 | 0.63 | 1.00 |  |  |  |  |  |  |  |  |  |
| Q6. Work/daily routine | 0.73 | 0.66 | 0.62 | 0.74 | 0.70 | 1.00 |  |  |  |  |  |  |  |  |
| Q7. Social activities and hobbies | 0.65 | 0.60 | 0.65 | 0.68 | 0.75 | 0.79 | 1.00 |  |  |  |  |  |  |  |
| Q8. Needing help | 0.61 | 0.56 | 0.55 | 0.72 | 0.61 | 0.72 | 0.67 | 1.00 |  |  |  |  |  |  |
| Q9. Sleep | 0.60 | 0.72 | 0.48 | 0.60 | 0.54 | 0.62 | 0.59 | 0.61 | 1.00 |  |  |  |  |  |
| Q10. Fatigue/low energy | 0.57 | 0.55 | 0.52 | 0.56 | 0.58 | 0.63 | 0.61 | 0.61 | 0.62 | 1.0 |  |  |  |  |
| Q11. Emotional well-being | 0.60 | 0.55 | 0.51 | 0.59 | 0.57 | 0.65 | 0.63 | 0.62 | 0.61 | 0.70 | 1.00 |  |  |  |
| Q12. Understanding | 0.12 | 0.12 | 0.10 | 0.13 | 0.11 | 0.15 | 0.10 | 0.12 | 0.11 | 0.11 | 0.15 | 1.00 |  |  |
| Q13. Self-efficacy | 0.42 | 0.38 | 0.31 | 0.38 | 0.35 | 0.44 | 0.39 | 0.40 | 0.37 | 0.38 | 0.43 | 0.46 | 1.00 |  |
| Q14. Overall impact | 0.78 | 0.71 | 0.58 | 0.67 | 0.65 | 0.76 | 0.71 | 0.67 | 0.68 | 0.65 | 0.69 | 0.18 | 0.51 | 1.00 |

Supplementary Figure S4. Inter-item polychoric correlation coefficients of individual items in the MSKHQ. This table shows the strength of relationship between items, where higher values indicate stronger associations between questions measuring similar concepts (i.e. musculoskeletal health).

|  | MSKHQ | HAQ-II | PHQ-2 & GAD-2 combined total | DAS-28 | TJC | SJC | PGH | ESR | CRP | ESR (log) | CRP (log) |
| --- | --- | --- | --- | --- | --- | --- | --- | --- | --- | --- | --- |
| MSKHQ | 1.00 |  |  |  |  |  |  |  |  |  |  |
| HAQ-II | -0.79 | 1.00 |  |  |  |  |  |  |  |  |  |
| PHQ-2 & GAD-2 combined total | -0.66 | 0.59 | 1.00 |  |  |  |  |  |  |  |  |
| DAS-28 | -0.42 | 0.46 | 0.28 | 1.00 |  |  |  |  |  |  |  |
| TJC | -0.33 | 0.34 | 0.24 | 0.76 | 1.00 |  |  |  |  |  |  |
| SJC | -0.22 | 0.25 | 0.14 | 0.68 | 0.64 | 1.00 |  |  |  |  |  |
| PGH | -0.43 | 0.40 | 0.31 | 0.63 | 0.38 | 0.31 | 1.00 |  |  |  |  |
| ESR | -0.20 | 0.28 | 0.13 | 0.58 | 0.15 | 0.22 | 0.18 | 1.00 |  |  |  |
| CRP | -0.19 | 0.26 | 0.12 | 0.41 | 0.16 | 0.24 | 0.17 | 0.59 | 1.00 |  |  |
| ESR (log) | -0.19 | 0.27 | 0.10 | 0.65 | 0.14 | 0.22 | 0.19 | 0.88 | 0.49 | 1.00 |  |
| CRP (log) | -0.21 | 0.30 | 0.12 | 0.51 | 0.19 | 0.27 | 0.20 | 0.61 | 0.81 | 0.61 | 1.00 |

Supplementary Figure S5. Concurrent validity of the MSKHQ against other measures. Pearson correlation coefficients shown. Higher values indicate better concurrent validity, meaning the MSKHQ correlates well with other established measures of similar concepts. MSKHQ: Musculoskeletal health Questionnaire, HAQ-II: Health Assessment Questionnaire-II, PHQ-2: Patient Health Questionnaire, GAD-2: Generalised Anxiety Disorder, DAS-28: Disease Activity Score-28, TJC: Tender Joint Count, SJC: Swollen Joint Count, PGH: Patient Global Health, ESR: Erythrocyte Sedimentation Rate, CRP: C-reactive Protein, ESR(log): Log transformation of the ESR value, CRP(log): Log transformation of the CRP value.


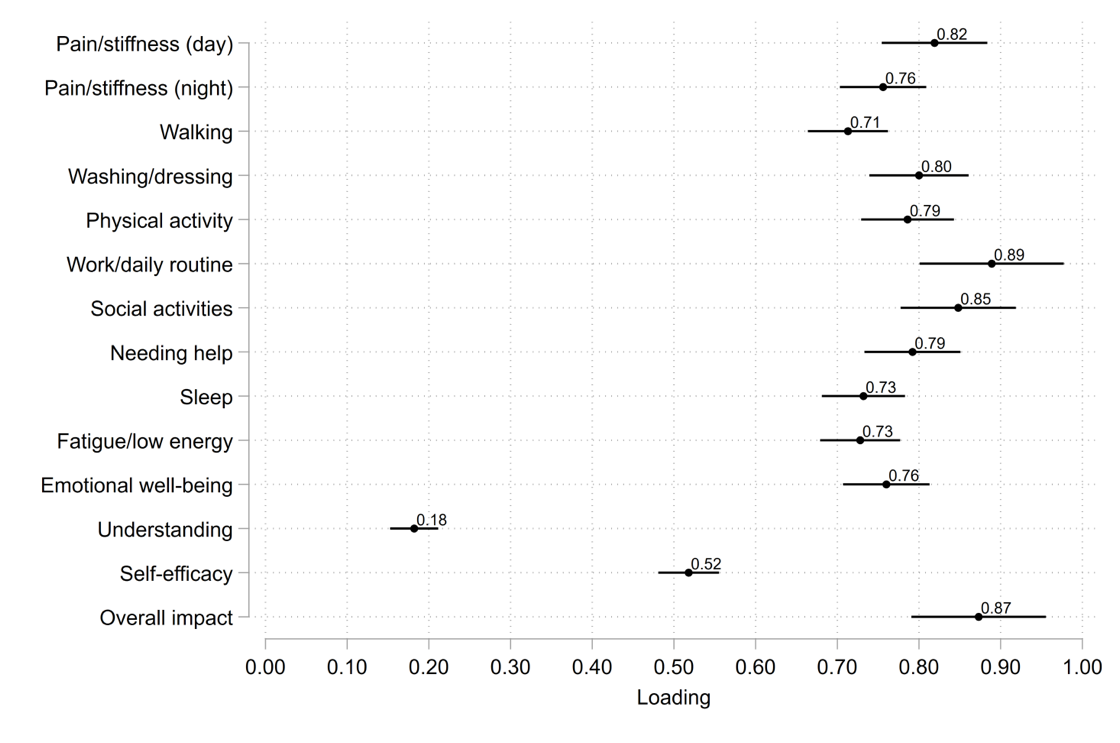


Supplementary Figure S6: Factor loadings of the MSKHQ individual items. This figure shows how strongly each item is related to the underlying factor it is meant to measure, where higher values mean the item is a better indicator of that factor.


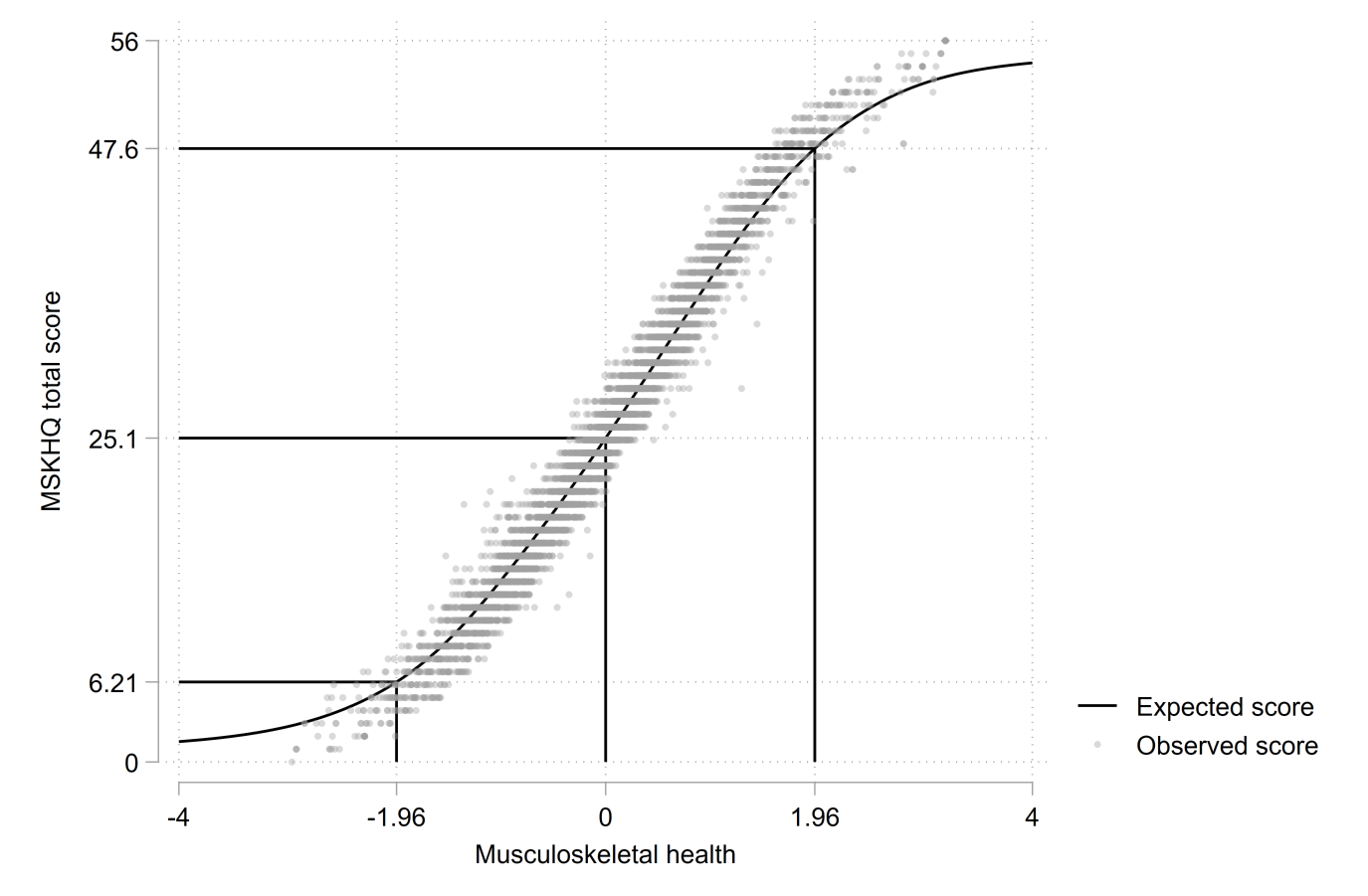


Supplementary Figure S7. Test characteristics curve measuring the relationship between the MSKHQ total score (y-axis) and the latent variable of musculoskeletal health (x-axis)


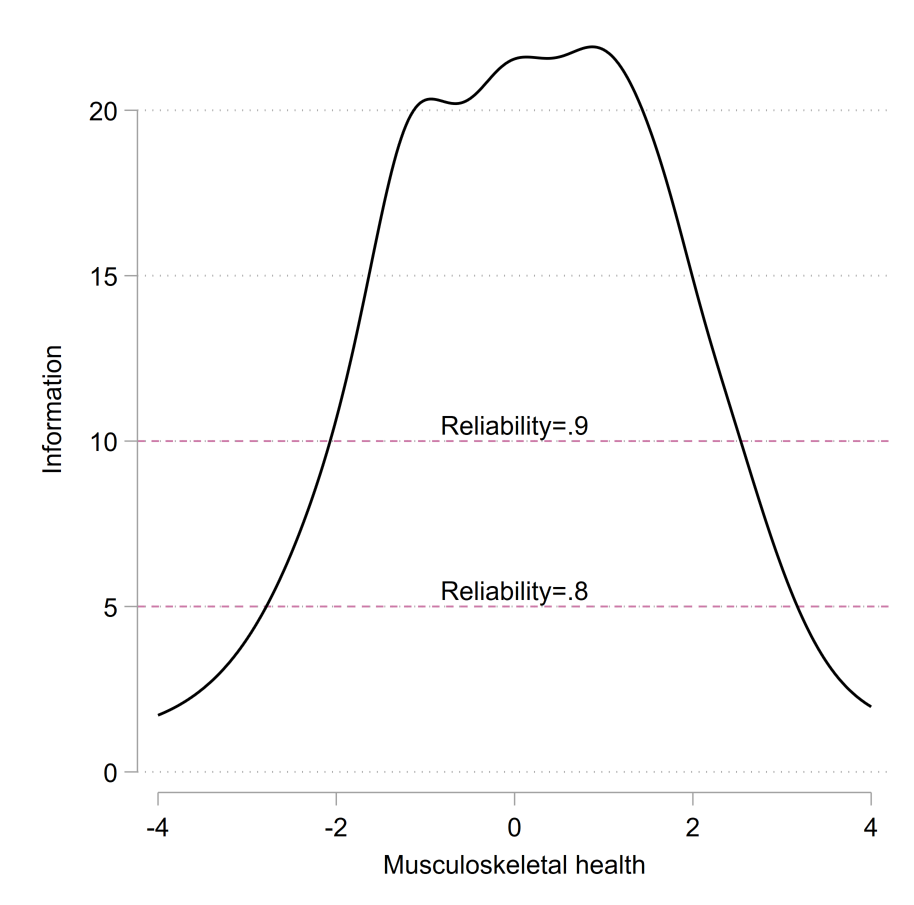


Supplementary Figure S8. Test information curve for the MSKHQ. The y-axis assesses reliability (precision) of the scale used to assess the latent variable of musculoskeletal health (x-axis). The dashed lines represent levels of test information where the reliability (Cronbach’s alpha) is 0.8 and 0.9. There is high reliability between around 3 standard deviations from the mean in both directions.


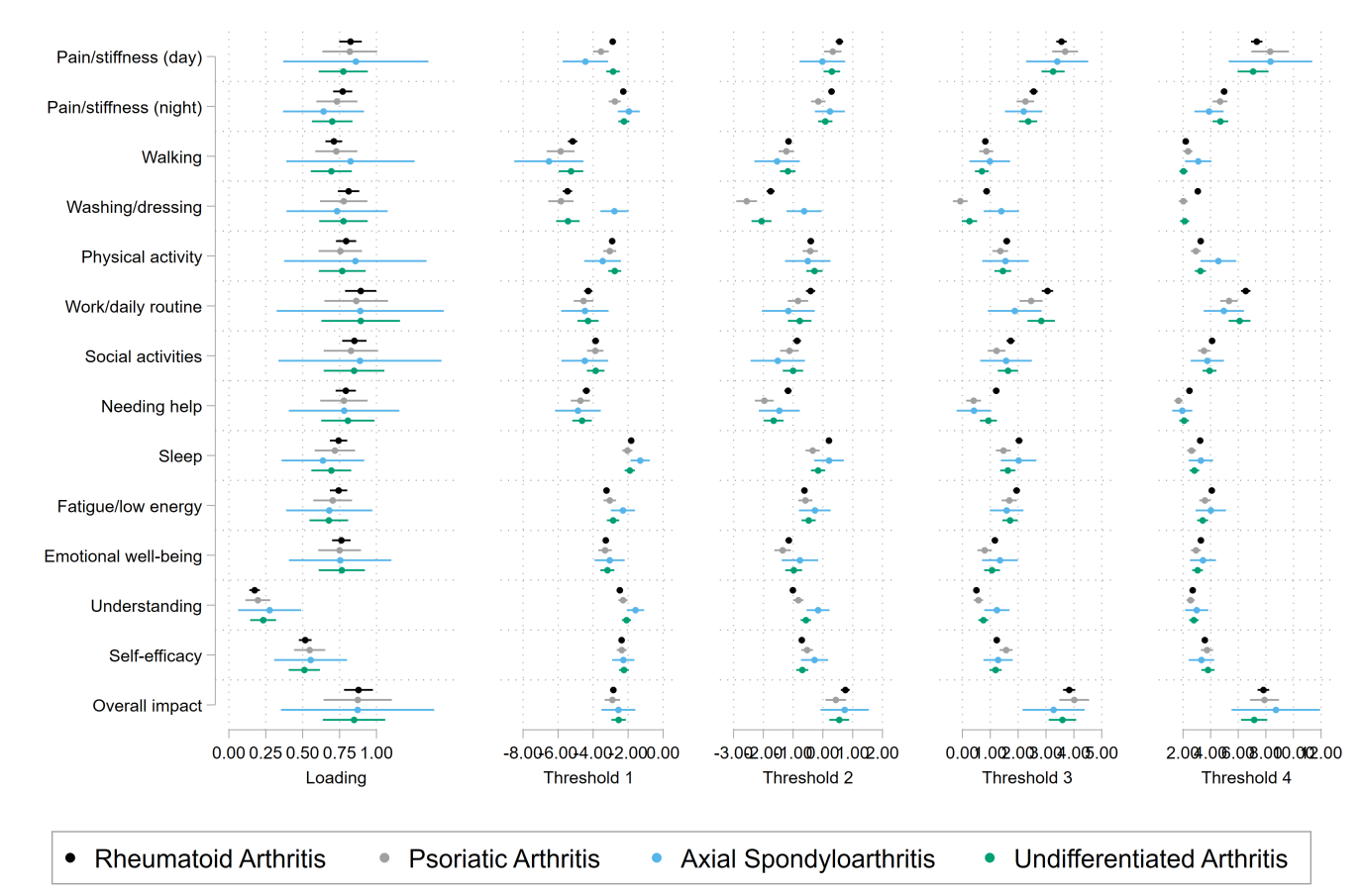


Supplementary Figure S9. Forest plot of the MSKHQ items on the y-axis, with the x-axis representing the loading (strength of the relationship between each item and the latent musculoskeletal health variable) and thresholds for each of the inflammatory arthritis (IA) subtypes. This helps compare how well each item performs across the IA subtypes.


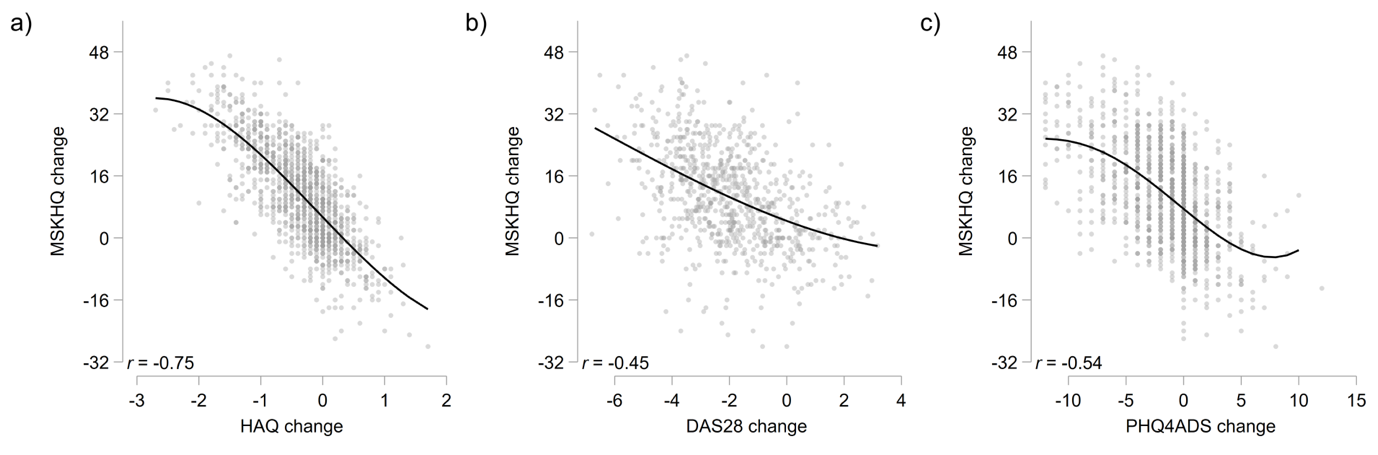


Supplementary Figure S10: Responsiveness of the MSKHQ versus a) HAQ-II, b) DAS-28, c) distress. This figure shows how well the MSKHQ detects change over time in relation to these other established tools.

| Working diagnosis | n | Mean | SD | alpha | MCID_SEM_ | MCID_⅓SD_ |
| --- | --- | --- | --- | --- | --- | --- |
| Rheumatoid Arthritis | 1283 | 32.0 | 11.3 | 0.94 | 3.6 | 3.8 |
| Psoriatic Arthritis | 188 | 30.1 | 12.1 | 0.94 | 3.8 | 4.0 |
| Axial Spondyloarthritis | 17 | 30.5 | 11.1 | 0.94 | 3.5 | 3.7 |
| Undifferentiated Arthritis | 175 | 32.1 | 11.3 | 0.93 | 3.6 | 3.8 |
| Total | 1663 | 31.8 | 11.4 | 0.94 | 3.6 | 3.8 |

Supplementary Figure S11. Means, SDs, reliability and MCID estimates for 12-item MSKHQ based on 12-month visit (full scale, range 0-48)
